# The Safety Attitudes Questionnaire: psychometric properties, benchmarking data, and emerging research

**DOI:** 10.1186/1472-6963-6-44

**Published:** 2006-04-03

**Authors:** John B Sexton, Robert L Helmreich, Torsten B Neilands, Kathy Rowan, Keryn Vella, James Boyden, Peter R Roberts, Eric J Thomas

**Affiliations:** 1 The University of Texas Center of Excellence for Patient Safety Research and Practice, The University of Texas – Houston Medical School, Houston, USA; 2 Center for AIDS Prevention Studies, University of California, San Francisco, USA; 3 Intensive Care National Audit & Research Centre, London, UK; 4 Royal Cornwall Hospital, Truro, Cornwall, TR1 3LJ, UK; 5 Medical Research Institute of New Zealand, Wellington, NZ; University of Texas – Houston Medical School, Division of General Medicine, Department of Medicine (EJT), USA

## Abstract

**Background:**

There is widespread interest in measuring healthcare provider attitudes about issues relevant to patient safety (often called safety climate or safety culture). Here we report the psychometric properties, establish benchmarking data, and discuss emerging areas of research with the University of Texas Safety Attitudes Questionnaire.

**Methods:**

Six cross-sectional surveys of health care providers (n = 10,843) in 203 clinical areas (including critical care units, operating rooms, inpatient settings, and ambulatory clinics) in three countries (USA, UK, New Zealand). Multilevel factor analyses yielded results at the clinical area level and the respondent nested within clinical area level. We report scale reliability, floor/ceiling effects, item factor loadings, inter-factor correlations, and percentage of respondents who agree with each item and scale.

**Results:**

A six factor model of provider attitudes fit to the data at both the clinical area and respondent nested within clinical area levels. The factors were: Teamwork Climate, Safety Climate, Perceptions of Management, Job Satisfaction, Working Conditions, and Stress Recognition. Scale reliability was 0.9. Provider attitudes varied greatly both within and among organizations. Results are presented to allow benchmarking among organizations and emerging research is discussed.

**Conclusion:**

The Safety Attitudes Questionnaire demonstrated good psychometric properties. Healthcare organizations can use the survey to measure caregiver attitudes about six patient safety-related domains, to compare themselves with other organizations, to prompt interventions to improve safety attitudes and to measure the effectiveness of these interventions.

## Background

Experts believe that hhhealthcare quality and safety must be investigated within the framework of systems and contextual factors in which errors and adverse events occur. [1-8] Vincent and colleagues describe several factors that influence clinical practice: organizational factors such as safety climate and morale, work environment factors such as staffing levels and managerial support, team factors such as teamwork and supervision, and staff factors such as overconfidence and being overly self assured. [8]

Healthcare provider attitudes about these and related factors are one component of an organization's safety culture. Influential organizations such as the UK National Health Service, the Joint Commission for the Accreditation of Healthcare Organizations, the Agency for Healthcare Research and Quality, and the U.S. National Quality Forum are encouraging the measurement of safety culture. This interest derives in part from the experience of other industries (nuclear power, naval aircraft carriers, NASA) that are known for their ability to reliably deal with risky processes. [9]

Despite considerable interest, there is limited psychometric and benchmarking data available for the surveys designed to measure these attitudes [10-13]. The aims of this paper are to present our experience with the University of Texas Safety Attitudes Questionnaire. We describe the survey's background, psychometric characteristics, provide benchmarking data, discuss how the survey can be used, and note emerging areas of research.

## Methods

### Terminology

Safety *culture *has been be defined as "the product of individual and group values, attitudes, perceptions, competencies, and patterns of behavior that determine the commitment to, and the style and proficiency of, an organization's health and safety management [13]." The SAQ elicits a snapshot of the safety culture through surveys of frontline worker perceptions. When using questionnaires to study group-level perceptions, the most appropriate term to use is climate (e.g., safety climate, or teamwork climate). Climates are more readily measurable aspects of safety culture (perceptions are part of both definitions) but surveys are generally not capable of measuring all other aspects of culture like behavior, values, and competencies. However, readers should be aware that some papers, organizations, and opinion leaders use the terms climate and culture interchangeably. We use the term *climate *where some may expect to see the phrase *culture of patient safety*.

Here we use clinical areas (a.k.a., work units, patient care areas, nursing units) as the group-level of interest. By testing the psychometrics of the SAQ at the individual level and the clinical area level, we can test the appropriateness of conceptualizing patient safety issues at the clinical area level, because clinical areas are generally associated with managers, geographical locations, and specific clinical and operational outcomes.

### Lineage and conceptual background of the Safety Attitudes Questionnaire (SAQ)

The Safety Attitudes Questionnaire (SAQ) is a refinement of the Intensive Care Unit Management Attitudes Questionnaire, [14,15] which was derived from a questionnaire widely used in commercial aviation, the Flight Management Attitudes Questionnaire (FMAQ). [16,17] The FMAQ was created after researchers found that most airline accidents were due to breakdowns in interpersonal aspects of crew performance such as teamwork, speaking up, leadership, communication, and collaborative decision making. The FMAQ measures crew member attitudes about these topics.

Because 25% of the FMAQ items demonstrated utility in medical settings in terms of the subject covered and factor loadings, they were retained on the SAQ, The new SAQ items were generated by discussions with healthcare providers and subject matter experts. In addition, we relied upon two conceptual models to decide which items to include: Vincent's framework for analyzing risk and safety [8] and Donabedian's conceptual model for assessing quality [18] This generated a pool of over 100 new items covering four themes: safety climate, teamwork climate, stress recognition, and organizational climate. Items were evaluated through pilot testing and exploratory factor analyses. This phase of survey development consistently yielded 6 factor-analytically derived attitudinal domains containing 40 items from the survey (two, three, four, and five factor structures were less robust). Three of the targeted themes, safety climate, teamwork climate, and stress recognition, emerged as factors. In particular, safety climate and stress recognition are conceptually quite similar to their counterparts in aviation. [19] The fourth targeted theme, organizational climate, consistently emerged as three distinct but related factors, perceptions of management, working conditions, and job satisfaction. Organizational climate plays a decisive role in setting the preconditions for success or failure in managing risks [3,4,20] , and we therefore retained these three factors as part of safety attitude assessment. An additional 20 items were retained because they were deemed interesting and valuable to the unit managers and senior hospital leadership to whom we reported the results of our pilot studies.

**Figure 1:**
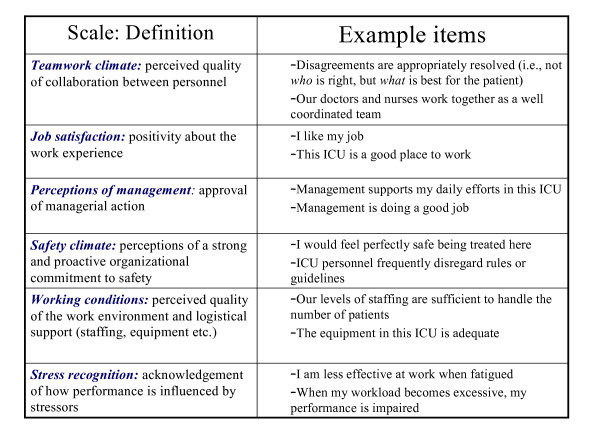
SAQ factor definitions and example items.

The SAQ has been adapted for use in intensive care units (ICU) [15,21] , operating rooms (OR), general inpatient settings (medical ward, surgical ward, etc.), and ambulatory clinics. For each version of the SAQ, item content is the same, with minor modifications to reflect the clinical area. For example, "In this ICU, it is difficult to discuss mistakes," vs. "In the ORs here, it is difficult to discuss mistakes." The SAQ elicits caregiver attitudes through the 6 factor analytically derived climate scales: teamwork climate; safety climate; job satisfaction; perceptions of management; working conditions; and stress recognition (Figure 1).

The SAQ is a single page (double sided) questionnaire with 60 items and demographics information (age, sex, experience, and nationality). The questionnaire takes approximately 10 to 15 minutes to complete. Each of the 60 items is answered using a five-point Likert scale (Disagree Strongly, Disagree Slightly, Neutral, Agree Slightly, Agree Strongly). Some items are negatively worded. There is also an open-ended section for comments: "What are your top three recommendations for improving patient safety in this clinical area?" Each version of the SAQ in the current study includes a "Collaboration and Communication" section, where respondents are asked to indicate the quality of collaboration and communication they have experienced with each of the types of providers in their clinical area (e.g., Staff Surgeons, Surgical Residents, Staff Anesthesiologists, OR Nurses, etc.) using a five-point Likert scale (Very Low, Low, Adequate, High, Very High).

### Chronology of the Safety Attitudes Questionnaire administrations

Early survey development, pilot studies, and exploratory factor analyses were conducted in four USA critical care sites. [14,15] This work lead to a six-factor solution using 40 of the 60 items, [21] and set the stage for the subsequent survey administrations reported in the current study. The data presented here came from six administrations (Table 1) of the SAQ between 2000 and 2003, totalling 203 sites (in the discussion we briefly note results of more recent survey administrations led by other investigators). We conducted further pilot testing of the SAQ for the United Kingdom and New Zealand, but aside from simple translations (e.g., USA Attendings and Residents became UK Consultants and Registrars, respectively) there were no substantial revisions. The first non-pilot version of the SAQ was administered in 106 United Kingdom (UK) ICUs. The second administration took place in 20 New Zealand (NZ) ICUs. The subsequent administrations occurred in the following sequence: 11 USA Inpatient settings, 2 USA OR settings, 11 USA Ambulatory Clinics, and 53 USA ICUs.

### Participants

To qualify for inclusion, both full- and part-time staff had to have worked in the unit (including those not based in the unit, but with a significant work commitment to it) for at least one month prior to administration of the questionnaire. The "rule of thumb" we applied was that all personnel within a clinical area who either influence or are influenced by the "working environment" in that clinical area were invited to participate (e.g., Attendings/Staff Physicians, Resident Physicians, Registered Nurses, Charge Nurses, Pharmacists, Respiratory Therapists, Technicians, Ward Clerks, Other:_____________). Response was voluntary, and administration techniques included hand-delivery, meeting administrations, and in-house mailing administrations.

### Data management and processing

SAQs were read into an OpScan8^® ^OMR scanner using ScanTools^® ^software, producing a tab-delimited file, which was converted into an SPSS Version 11.5 file for analysis. The Likert scale (1 = Disagree Strongly, 2 = Disagree Slightly, 3 = Neutral, 4 = Agree Slightly, 5 = Agree Strongly) was used to score each of the 60 items. Negatively worded items were reverse scored so that their valence matched the positively worded items.

### Data analysis

Each clinical area possesses a unique social fabric, leading respondents who work within the same clinical area to respond more similarly than respondents who are members of different clinical areas. Consequently, it is important to control for the non-independence of responses gathered from the same clinical area via performing analyses that address the multilevel nature of the data in order to obtain accurate model test statistics and scale reliability estimates. Therefore, we fit the hypothesized six-factor model via multilevel confirmatory factor analysis using Mplus version 2.12. [22] We used the entire sample of respondents in order to make the maximum number of clinical areas (n = 203) available for parameter estimation at the clinical area level. To evaluate the overall fit of each model to the data, we used the Mplus MLR chi-square test of model fit that is robust to non-normal data. [23] This estimator uses White's sandwich-based method to yield test statistics that are robust to misspecification of the model's factor structure and non-normal input data [24]. While this robust estimator yields superior results compared to standard maximum likelihood when input data are non-normal, the chi-square test of absolute model fit can still be sensitive to trivial misspecifications in the model's structure, however, so we also evaluated the following descriptive measures of model fit: the standardized root mean residual (SRMR) [25] , the Comparative Fit Index (CFI) [26] , and the Root Mean Square Error of Approximation (RMSEA) [27] using the recommended cutoff values of .90 for the CFI and related incremental fit indices, .08 for the RMSEA, and .10 for the SRMR [28]. We initially fit a six factor multi-level confirmatory factor analysis model that contained the 40 items retained in previous studies that explored the SAQ's construct validity [21]. Items with weak factor-item associations at the clinical area level or individual level were then deleted sequentially via a backward elimination procedure until satisfactory model fit was attained.

For purposes of consistency and to display separate reliability results for clinical areas and for individuals nested within clinical areas, we computed coefficient alpha values in Mplus using the structural equation modeling-based approach of Miller [29] and Raykov [30].

Once satisfactory model fit was obtained, we used the model results to compute composite scale reliability using Raykov's ñ coefficient. Coefficient alpha, the usual statistic used to estimate scale reliability, assumes that all items' factor loadings are identical, a restrictive assumption that biases scale reliability estimates [30]. Raykov's ñ relaxes this assumption, yielding more accurate reliability estimates. Moreover, coefficient alpha is limited to single-level analyses whereas ñ has recently been extended to incorporate multilevel analysis scenarios of the type presented here [31] Accordingly, we report ñ below as the scale reliability estimate for the SAQ.

### Terminology and interpretation

For ease of interpretability, we conducted analyses on mean scores, but also present percent agreement to facilitate understanding of the items and scales. The percentage of respondents within a clinical area reporting "agree slightly" or "agree strongly" for each of the items within a given scale were charted as the percent positive. When individual attitudes are aggregated by clinical area, the SAQ provides a snapshot of the climate in a given clinical area (i.e., one attitude is an opinion, but the aggregate attitudes of everyone in a clinical area is climate). Attitudinal questionnaires are also informative in organization-wide assessments of climate, but it is important to interpret organization-wide results at the work-unit or clinical area level as well, due to the high degree of variability between clinical areas within the same organization [32]. Variability within an organization is not unique to healthcare settings, as we have found that there is generally more variability within an airline between fleets (types of aircraft) and departments, than there is between organizations. For clarification of terms, we use the phrases "clinical area" and "site" to refer to all of the respondents from a given ICU, OR, Inpatient Ward, or Ambulatory Clinic.

## Results

### SAQ administrations

The overall SAQ response rate was 67.0% (10,843 out of 16,184 questionnaires), with a range of 65.7% to 72.2% across administrations. Response rates and floor/ceiling effects for each scale are presented in Table 1, by administration.

Table 2 presents the SAQ factors' descriptive data by administration, including overall means, minimum and maximum clinical area means within an administration, and overall standard deviations. Incomplete data at the item level was approximately 1.5% overall (Table 3). Descriptive analyses of individual items should not be appreciably affected by such a small amount of incomplete data. [33] There was substantial variability across the 203 clinical areas at the item level. In total, for example, one out of five respondents reported that it is difficult to speak up if they perceive a problem with patient care, but at the clinical area level, the percent of respondents who agree ranged from 0% to 50%. In other words, zero respondents reported difficulty speaking up in some clinical areas, while in other clinical areas, half of the caregivers reported difficulty speaking up.

### Safety Attitudes Questionnaire: factor structure and multi-level modeling

To assess the fit of the expected six factor structure to the data, we fit a sequence of six factor multi-level confirmatory factor analysis models to the survey data. [34,35] Of the original 10,843 cases, 10,810 were associated with an identifiable clinical area; there were 203 available clinical areas for these analyses.

The SAQ with six factors and 40 items (plus 20 additional items) was used in all the administrations reported here. However our analysis for this paper used a more rigorous multi-level confirmatory factor analysis and prompted us to drop ten items to attain satisfactory model fit for the majority of fit indices. The fit of the final model containing the 30 remaining items was generally satisfactory: χ^2^(784) = 10,311.27, p < .0001; CFI = .90, RMSEA = .03, SRMR (between clinical areas) = .17, and SRMR (within clinical areas) = .04. Standardized factor loadings at the clinical area and individual levels for the 30 retained items from the final multi-level confirmatory factor analysis appear in Table 3. The correlations between the factors are shown in Table 4

### Reliability assessments

Composite scale reliability for the SAQ was assessed via Raykov's *ρ *coefficient. The *ρ *value for the SAQ in this sample was .90, indicating strong reliability of the SAQ. Overall, this finding, in conjunction with the multi-level factor analyses demonstrated that the SAQ has good psychometric properties. Also, anecdotal evidence from respondents during feedback presentations indicates that the SAQ items are in fact assessing topics of importance to front-line personnel.

### Benchmarking climate

**Figure 2:**
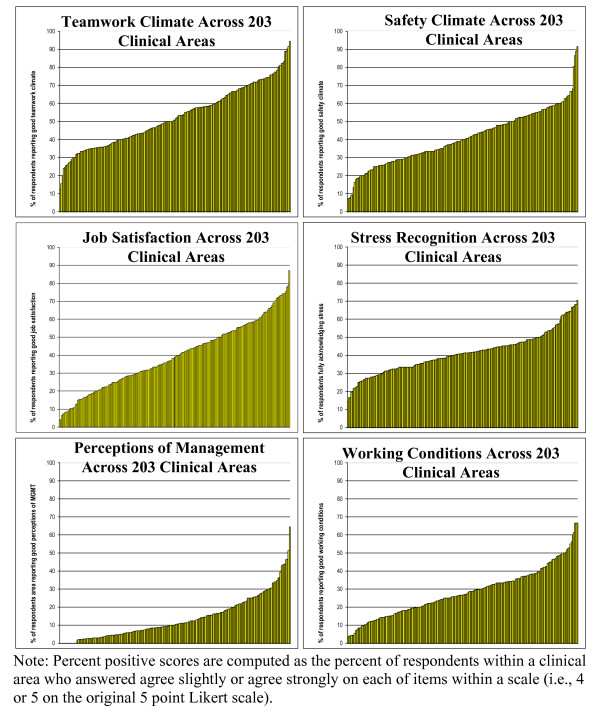
Distribution of percent positive scores for the 203 clinical areas.

The percentage of respondents within a clinical area reporting "agree slightly" or "agree strongly" for each of the items within a given scale were charted as the percent positive for each SAQ factor. The six SAQ distributions in Figure 2 demonstrate the variability in percent positive SAQ scores across the 203 clinical areas in the present study.

## Discussion

The SAQ is a psychometrically sound instrument for assessing six safety-related climate domains by systematically eliciting input from front-line caregivers. The SAQ can be used to meet the increasing demand for safety climate (often called safety culture) assessment at the clinical area level. For comparison purposes, those interested may use the 203 clinical areas reported here, as they demonstrated substantial variability in teamwork climate, safety climate, job satisfaction, stress recognition and working conditions. We found substantial variability in teamwork climate, safety climate, job satisfaction, stress recognition and working conditions. The item descriptives (Table 3), together with the percent positive distributions (Figure 2), and the administration-level descriptives (Table 1 and Table 2), serve as benchmarking data for the SAQ. Examination of Table 4 shows that the 6 factors have lower correlations at the clinical area level than at the individual respondent level, indicating that the 6 factors are more diagnostic (share less variance with each other) when used at the clinical area level.

For example, institutions like the Memorial Hermann Healthcare System, Ascension Health, and Johns Hopkins Hospital use the SAQ to assess safety climate hospital-wide, (at the clinical area level) annually. These clinical areas benchmark their climate against other units in their institutions and against themselves. Strengths and weaknesses in a given clinical area (relative to comparison data) can be identified and appropriate interventions undertaken. For example, a poor teamwork climate would suggest collaborative rounds, [36] whereas a poor safety climate would suggest Leadership WalkRounds [37] or a Comprehensive Unit-based Safety Program [38]. When used in a pre-intervention/post-intervention methodology, the SAQ factors have demonstrated sensitivity to quality improvement interventions at Kaiser Permanente [39] and recent evidence from Johns Hopkins Hospital demonstrates that climate can be targeted and improved. These improvements are associated with reductions in medication errors and with shorter lengths of stay [38]. Recent data from the Keystone ICU collaborative of critical care units in Michigan demonstrated that critical care units with the highest scores on SAQ factors had the lowest subsequent blood-stream infection rates (personal communication: Peter Pronovost, June 2005).

Such examples of how the SAQ is used help provide information about convergent validity and construct validity, two important psychometric characteristics not analyzed in this study. For example, SAQ climate scores of critical care personnel are correlated with James Reason's Checklist for Assessing Institutional Resilience (CAIR) scores of middle-level managers in the same institutions. [40] In other words, an independent assessment of safety climate using a different instrument produced the expected convergent results. Also, analyses of the SAQ open ended comments for "What are your top three recommendations for improving patient safety in this clinical area?" provides a form of convergent validity when the content of comments is linked to the SAQ factor scores. For example, ICUs with poor teamwork climate scores had significantly more respondent comments regarding the need to improve communication, relative to ICUs with high teamwork climate scores. [41] Similarly, ICUs with high stress recognition scores made more recommendations regarding the need for increased staffing levels relative to low stress recognition ICUs (i.e., respondents who acknowledge the effects of stress on their performance were much more likely to identify the need for improved staffing levels).

Our results indicate that researchers should consider hospitals comprised of clinical areas to resemble corporations comprised of organizations, because the clinical areas appear to resemble what are typically considered organization-like unique climates. The multi-level model demonstrated that there is more variability between clinical areas than within clinical areas. In other words, context of care assessments appear to be more robust, meaningful, and interpretable at the clinical area level. Climate at the clinical area level is important as many clinical and operational outcomes are tracked at the clinical area level (e.g., catheter related blood stream infections in intensive care units), and it is easier to target clinical area level improvements than hospital wide improvements. We see the focus on clinical area level climate as a way to acknowledge the complexity of the systems in which caregivers work, rather than assuming monolithic hospital climates that lack diagnosticity of clinical area level issues.

The SAQ differs from other medical safety climate or "culture" surveys [10-13] in four respects: first, the SAQ has been more widely used for a longer period of time, so there is benchmarking data available and many of the challenges of longitudinal assessment have been encountered and addressed; second, a larger amount of psychometric data is available for the SAQ; and third, the SAQ maintains continuity with its predecessor (the FMAQ) – a traditional human factors survey with a 20 year history in aviation. [16,17] The availability of benchmarking data in the public domain enables organizations to evaluate their own climate data. Also, preserving item continuity with other high-reliability industries allows for comparisons between professions [14] , and assists with the search for universal human factors issues across professions.

### Limitations

The SAQ demonstrates generally good psychometric properties, though a number of the factor loadings at the clinical area level were smaller in magnitude than the corresponding factor loadings at the individual level. Not surprisingly, the standardized root mean residual (SRMR) model fit statistic at the clinical area level was larger than desirable, indicating that further scale refinement could result in stronger factor loadings and a better fitting model at the clinical area level without sacrificing integrity of measurement at the individual caregiver level. As noted above, a number of studies have linked SAQ factor scores to important clinical outcomes [38] and other instruments that are used in healthcare. [40] Nonetheless, further research on the relationship between SAQ factors and other variables such as staff turnover, patient morbidity, length of stay, and errors is needed.

Lastly, due to the limited scope of this study, it was not possible to assess factor structure invariance across countries, job categories of respondents, or other stratification variables. At a minimum, we demonstrated the validity of the SAQ in a large heterogeneous sample made up of many different healthcare provider types, clinical areas of various acuity levels, countries of origin, hospital types, gender, experience level, etc., in hopes that the results will generalize to a wide variety of healthcare providers.

## Conclusion

It is possible to reliably and meaningfully measure caregiver attitudes and perceptions relevant to the safety of healthcare. Use of the SAQ to assess climate in clinical areas will allow valid comparisons between hospitals, patient care areas, and types of caregivers, and tracking of change over time. We can and should do more to tap into the wisdom and perspective of the frontline caregivers regarding the contexts in which they deliver care. Versions of the SAQ, as well as the SAQ Users Manual and additional benchmarking data can be downloaded from our website. [42]

## Abbreviations

SAQ – Safety Attitudes Questionnaire

ICU – Intensive Care Unit

OR – Operating Room

UK – United Kingdom

USA – United States of America

FMAQ – Flight Management Attitudes Questionnaire

## Competing interests

The author(s) declare that they have no competing interests.

## Authors' contributions

JBS – conception, design, acquisition of data, analysis and interpretation of data, drafting of manuscript, revising manuscript, final approval.

RLH – conception, design, interpretation of data, drafting of manuscript, revising manuscript, final approval.

TBN – analysis and interpretation of data, revising manuscript, final approval

KR – conception, design, acquisition of data, analysis and interpretation of data, revising manuscript, final approval.

KV – conception, design, acquisition of data, analysis and interpretation of data, revising manuscript, final approval.

JB – conception, design, acquisition of data, analysis and interpretation of data, revising manuscript, final approval.

PRR – conception, design, acquisition of data, analysis and interpretation of data, revising manuscript, final approval.

EJT – conception, design, acquisition of data, analysis and interpretation of data, drafting of manuscript, revising manuscript, final approval.

## Pre-publication history

The pre-publication history for this paper can be accessed here:



## Figures and Tables

**Table 1:** SAQ administration response rates and floor and ceiling effects

							**percent floor/percent ceiling**			
**SAQ Version-Country**	**# sites**	n returned	n administered	**response rate**	teamwork climate	safety climate	perceptions of management	job satisfaction	working conditions	stress recognition

ICU-UK	106	4856	7390	65.7%	.08/6.41	.02/1.77	1.15/.27	.37/2.10	.31/1.55	.58/3.53
ICU-NZ	20	761	1054	72.2%	.26/2.50	.00/1.32	1.19/.13	.92/1.18	.26/.66	.13/8.54
ICU USA	53	3029	4415	68.6%	.10/3.11	.00/2.28	1.06/1.72	.13/5.09	.40/2.88	.73/6.48
Inpatient-USA	11	1531	2330	65.7%	.13/4.02	.07/4.52	1.32/3.43	.39/7.72	.86/3.29	.86/7.83
OR-UK/NZ	2	385	575	67.0%	.00/1.04	.00/.52	2.86/.00	1.04/1.04	1.30/.78	.26/14.58
Ambulatory Clinic-USA	11	281	420	67.0%	.00/14.23	.00/4.98	.71/2.85	.00/12.10	2.50/6.79	6.43/5.36
OVERALL	203	10843	16184	67.0%	.10/4.90	.00/2.30	1.20/1.20	.40/3.90	.50/2.20	.80/5.80

This chart details the number of clinical areas within an SAQ administration, the number of respondents, and the response rate. Note: percentage floor/percentage ceiling = the percent of scores at the extremes of the scaling range

**Table 2:** SAQ factor descriptives: overall means (minimum and maximum); and overall standard deviations (overall score sample sizes come from n returned in Table 1)

Overall mean (min mean for the clinical area – max mean for the clinical area)/overall s.d.						
**SAQ version-country**	teamwork climate	safety climate	perceptions of management	job satisfaction	working conditions	stress recognition
ICU-UK	74.3 (55.9 – 87.2)/18.3	67.7 (55.7 – 84.8)/16.7	44.6 (26.8 – 59.5)/19.0	60.7 (40.4 – 77.1)/21.2	59.6 (38.5 – 75.5)/19.9	64.2 (56.7 – 72.0)/19.6
ICU-NZ	67.9 (45.5 – 78.6)/18.8	63.8 (50.0 – 72.4)/17.4	45.3 (26.4 – 57.7)/20.0	59.9 (41.0 – 73.1)/21.8	53.7 (38.5 – 64.8)/19.9	71.7 (62.1 – 81.0)/19.6
ICU-USA	65.7 (52.3 – 78.6)/19.0	68.8 (58.6 – 77.9)/17.4	54.1 (33.9 – 75.5)/22.4	68.6 (42.7 – 89.1)/22.3	58.3 (45.4 – 72.5)/21.4	67.2 (57.4 – 77.8)/21.2
inpatient-USA	64.3 (62.3 – 74.1)/16.6	60.5 (63.4 – 76.8)/16.0	38.3 (50.1 – 67.8)/18.7	59.6 (61.9 – 77.7)/20.5	49.2 (50.9 – 64.1)/19.5	74.4 (55.9 – 77.2)/20.2
OR-UK	71.7 (62.1 – 67.3)/22.6	69.6 (57.9 – 64.0)/18.9	47.6 (37.1 – 39.8)/22.2	70.1 (55.4 – 65.2)/22.1	57.5 (48.9 – 49.6)/25.4	54.7 (72.7 – 76.8)/26.6
ambulatory -USA	69.7 (62.9 – 84.4)/17.0	69.9 (60.1 – 84.7)/16.2	55.3 (41.0 – 63.8)/21.9	70.6 (57.0 – 84.7)/20.2	61.6 (44.8 – 79.6)/20.2	66.7 (41.6 – 69.7)/21.1

**Table 3:** SAQ item descriptives used for benchmarking

teamwork climate	% item missing data	mean (sd)	% agree (min agree-max agree)	% disagree (min disagree-max disagree)	factor loading (between)	factor loading (within)
It is easy for personnel in this ICU to ask questions when there is something that they do not understand.	**1.4**	4.17 (.96)	81 (42–100)	7 (0–35)	0.91	0.65
I have the support I need from other personnel to care for patients.	**2.2**	3.97 (.99)	74 (33–98)	9 (0–43)	0.90	0.65
Nurse input is well received in this ICU.	**1.6**	3.98 (1.05)	73 (24–100)	10 (0–55)	0.76	0.61
In this ICU, it is difficult to speak up if I perceive a problem with patient care.	**2.0**	2.40 (1.21)	22 (0–50)	60 (9–100)	-0.86	-0.42
Disagreements in this ICU are resolved appropriately (i.e., not *who *is right, but *what *is best for the patient)	**1.7**	3.53 (1.10)	57 (23–85)	18 (0–55)	0.85	0.61
The physicians and nurses here work together as a well-coordinated team.	**1.6**	3.78 (1.07)	68 (26–98)	14 (0–52)	0.76	0.63
safety climate						
The culture in this ICU makes it easy to learn from the errors of others.	**1.8**	3.95 (1.01)	72 (33–100)	10 (0–33)	0.94	0.59
Medical errors are handled appropriately in this ICU.	**2.2**	3.45 (1.06)	51 (14–92)	17 (0–57)	0.83	0.59
I know the proper channels to direct questions regarding patient safety in this ICU.	**1.6**	3.83 (1.01)	64 (24–100)	9 (0–38)	0.78	0.43
I am encouraged by my colleagues to report any patient safety concerns I may have	**1.4**	4.08 (.94)	78 (48–100)	7 (0–26)	0.94	0.60
I receive appropriate feedback about my performance.	**0.9**	3.20 (1.23)	46 (5–77)	31 (0–76)	0.73	0.58
I would feel safe being treated here as a patient.	**1.2**	4.05 (1.04)	75 (36–100)	9 (0–42)	0.54	0.62
In this ICU, it is difficult to discuss errors.	**1.6**	2.53 (1.13)	20 (0–46)	52 (21–92)	-0.69	-0.40
job satisfaction						
This hospital is a good place to work.	**0.9**	3.73 (1.08)	63 (5–100)	13 (0–59)	0.99	0.81
I am proud to work at this hospital.	**0.8**	3.78 (1.07)	62 (16–100)	11 (0–50)	0.97	0.80
Working in this hospital is like being part of a large family.	**0.5**	3.10 (1.30)	42 (0–94)	33 (0–80)	0.91	0.69
Moral in this ICU area is high.	**1.4**	2.96 (1.25)	39 (4–83)	37 (0–78)	0.69	0.61
I like my job.	**0.3**	4.37 (.88)	85 (61–100)	5 (0–18)	0.73	0.57
stress recognition						
When my workload becomes excessive, my performance is impaired.	**1.2**	3.83 (1.13)	72 (29–100)	15 (0–53)	0.96	0.60
I am more likely to make errors in tense or hostile situations.	**1.2**	3.74 (1.16)	67 (30–88)	17 (0–50)	0.74	0.57
Fatigue impairs my performance during emergency situations (e.g., emergency resuscitation, seizure).	**3.5**	3.00 (1.28)	40 (6–79)	36 (13–76)	0.51	0.45
I am less effective at work when fatigued.	**1.1**	3.97 (1.03)	77 (38–96)	11 (0–30)	0.92	.75
perceptions of management						
Hospital management does not knowingly compromise the safety of patients.	**1.9**	3.21 (1.22)	41 (9–87)	27 (5–91)	0.71	0.58
Hospital administration supports my daily efforts.	**0.8**	2.75 (1.15)	25 (0–93)	40 (0–100)	0.84	0.69
I am provided with adequate, timely information about events in the hospital that might affect my work.	**1.6**	3.16 (1.09)	42 (12–74)	27 (0–64)	0.76	0.52
The levels of staffing in this clinical area are sufficient to handle the number of patients	**1.7**	2.68 (1.34)	33 (0–85)	52 (4–96)	0.56	0.43
working conditions						
All the necessary information for diagnostic and therapeutic decisions is routinely available to me.	**2.3**	3.56 (1.08)	58 (17–90)	18 (0–67)	0.59	0.54
This hospital constructively deals with problem physicians and employees.	**1.7**	2.82 (1.12)	25 (0–83)	35 (0–80)	0.83	0.57
Trainees in my discipline are adequately supervised.	**2.7**	3.53 (1.17)	58 (10–100)	21 (0–63)	0.73	0.56
This hospital does a good job of training new personnel.	**1.1**	3.54 (1.18)	57 (16–96)	20 (0–61)	0.72	0.67

Table 3 Provides general descriptive information at the item level (likert scale: 1 = disagree strongly, 2 = disagree slightly, 3 = neutral, 4 = agree slightly, 5 = agree strongly): percent missing data; overall mean (standard deviation); overall percent agree (minimum agree-maximum Agree by clinical area); overall percent disagree (minimum disagree-maximum disagree by clinical area); standardized factor loadings at the between-area and within-area levels.

**Table 4:** SAQ factor intercorrelations

**factor**	**teamwork climate**	**safety climate**	**job satisfaction**	**stress recognition**	**perceptions of management**	**working conditions**
**teamwork climate**	1.00	.72	.33^a^	-.31	.26^a^	.79
**safety climate**	.94	1.00	.67	-.09^a^	.78	.95
**job satisfaction**	.76	.76	1.00	-.01^a^	.93	.66
**stress recognition**	-.12	-.12	-.14	1.00	-.09^a^	-.28^a^
**perceptions of management**	.69	.75	.82	-.17	1.00	.79
**working conditions**	.86	.91	.78	-.15	0.86	1.00

**Notes: Between-area correlations appear above the diagonal; within-area correlations appear below the diagonal. *N *= 10,810 respondents from 203 clinical areas. Factor intercorrelations were estimated in the final multilevel factor model using M *plus*. All correlations are *p *< .05, except^a^**.
